# Gnotobiotic Operations and Assembly for Development of Germ-Free Animal Model of Laser-Induced Choroidal Neovascularization

**DOI:** 10.1167/tvst.10.9.14

**Published:** 2021-08-13

**Authors:** Asadolah Movahedan, Hugo Barba, Melanie Spedale, Nini Deng, Donna Arvans, Urooba Nadeem, Vanessa Leone, Eugene B. Chang, Betty Theriault, Dimitra Skondra

**Affiliations:** 1 Department of Ophthalmology and Visual Sciences, The University of Chicago, Chicago, IL, USA; 2 Animal Resources Center, The University of Chicago, Chicago, IL, USA; 3 Department of Pathology, The University of Chicago, Chicago, IL, USA; 4 Section of Gastroenterology and Nutrition, Department of Medicine, University of Chicago, Chicago, IL, USA; 5 The Microbiome Center, University of Chicago, Chicago, IL, USA; 6 Department of Surgery, The University of Chicago, Chicago, IL, USA

**Keywords:** age-related macular degeneration, microbiome, choroidal neovascularization, germ-free, gnotobiotic, animal model

## Abstract

**Purpose:**

Compelling new evidence reveals a close link between the gut microbiome and the pathogenesis of neovascular age-related macular degeneration (nAMD). Germ-free (GF) animal models are the current gold standard for studying host the microbe interactions in vivo; yet, no GF animal models of nAMD are available today. This protocol describes gnotobiotic operations and assembly for a laser-induced choroidal neovascularization (CNV) model in GF mice to study the gut microbiome in neovascular AMD.

**Methods:**

We developed a step-wise approach to performing retinal laser photocoagulation in GF C57BL/6J mice that were bred and maintained at the gnotobiotic facility. Following a strict sterility protocol, we administered laser photocoagulation via an Argon 532-nm laser attached to a customized slit-lamp delivery system. Sterility was confirmed by weekly fecal cultures and reverse transcriptase–polymerase chain reaction.

**Results:**

The experiment was repeated twice at different time points using seven mice (14 eyes). Stool cultures and RT-PCR remained negative for 14 days post-procedure in all mice. Lectin immunostaining performed on choroidal flatmounts confirmed the presence of CNV lesions 2 weeks after laser treatment.

**Conclusions:**

We established a GF mouse model of nAMD with detailed guidelines to deliver retinal laser in GF mice maintaining sterility after the laser procedure.

**Translational Relevance:**

To our knowledge, this is the first protocol that describes a GF murine model of laser-induced CNV. In addition to nAMD, this animal model can be used to investigate host–microbial interactions in other eye diseases with laser-induced mouse models such as glaucoma and retinal vein occlusion.

## Introduction

Age-related macular degeneration (AMD) is the leading cause of blindness among adults over 55 years old.^1^^,^^2^ The clinicopathologic hallmark of neovascular AMD is choroidal neovascularization. AMD is a complex multifactorial disease in which genetic predisposition plays an important role, but disease development appears to be significantly affected by environmental and lifestyle factors such as smoking, obesity, and diet. However, the pathogenesis of AMD is still not fully understood.^3^^,^^4^ Emerging new studies have shown that the gut microbiome and in particular diet-induced changes of the gut microbiota may play an important role in the pathogenesis of AMD.^5^^–^^10^ Nonetheless, there is still a long road ahead to unravel the mechanisms of microbiome–host interactions involved in the development of this disease. This notion has opened up a new frontier for investigators to revisit previously described mechanisms such as inflammation, stress, immune response, and angiogenesis in their search for links to microbiome–host interactions and also to explore new pathways, biomarkers, and targets that may result in more effective approaches in the treatment and prevention of AMD.^6^^,^^9^^,^^10^

In the past decade, the role of the gut microbiome has been elucidated in health and several disease states. Current advancements in microbiome research are largely due to the development of new technologies and, in particular, animal models of an altered microbiome, specifically germ-free (GF) mouse models.^6^^,^^11^^–^^16^

Laser-induced choroidal neovascularization (CNV) is the most commonly used model for neovascular AMD. In this model, the lesions are created using laser photocoagulation to perforate the Bruch's membrane, resulting in neovascular response at the site of injury.^17^^,^^18^ This model has significantly changed our understanding of the development of neovascular AMD and has resulted in the discovery of the majority of current and emerging treatments for neovascular AMD.^19^^–^^21^

GF and antibiotic-treated animal models are the most common tools used to study the effects of the microbiome. GF mice are born and raised under special sterile conditions in plastic isolators within a gnotobiotic facility and thus lack any microorganisms^22^^–^^24^; in contrast, the antibiotic model is treated with broad-spectrum antibiotics and antimycotics to deplete microorganisms.^24^ Both models can cause biological changes such as enlarged cecum, changes in the immune cell populations, and alteration in bone density.^23^^–^^26^ However, there are major biologic differences between the two groups. GF mice are less susceptible to colonic inflammation, whereas the immune cell populations, specifically B-cells^27^ and dendritic cells,^26^^,^^28^^–^^30^ are reduced in antibiotic-treated mice. There are multiple disadvantages to antibiotic animal models. Antibiotics may influence coagulation and platelet aggregation by interfering with vitamin K metabolism.^27^^,^^31^^–^^35^ Oral administration of the antibiotics can alter the animal's food consumption and water intake, leading to dehydration and thus changing the desired dosages of the medications and altering the microbiome status.^36^^–^^39^ Likewise, strategies to administer these medications, such as oral gavage, are laborious and often do not completely eradicate all gut bacteria, and the animal can still harbor bacteria/microbes colonizing tissues such as skin and lungs that can interfere with experimental results.

Currently, there is no GF animal model of AMD, but we believe that there is an absolute need for such a model for microbiome research in AMD. This protocol describes the gnotobiotic operations and assembly of equipment to establish and sustain a laser-induced choroidal neovascularization model of GF mice that will allow for comprehensive study of the gut microbiome in neovascular AMD and retinal angiogenesis.

## Materials and Methods

### Facility

All experiments were performed at the Gnotobiotic Research Animal Facility (GRAF) at the University of Chicago. Standard gnotobiotic procedures and protocols, including the use of sterile isolators, sterile transfer cages, and sterilization cylinders, as well as handling and maintenance, have been described previously.^40^

### Animals

Seven C57BL/6J GF mice ages 8 to 16 weeks (five females and two males) underwent the procedures. Both eyes of each animal were lasered (14 eyes). We first performed the experiments with three animals and then repeated the protocol in a second set of four animals. All experiments were performed in accordance with the ARVO Statement for the Use of Animals in Ophthalmic and Vision Research and were in compliance with the Guide for the Care and Use of Laboratory Animals^41^ and were reviewed and approved by the University of Chicago Institutional Animal Care and Use Committee (ACUP# 72557). All measures were taken to ameliorate animal suffering per the ARVO statement. The University of Chicago is an American Association for Accreditation of Laboratory Animal Care–accredited institution with a public health service assurance on file with the Office of Laboratory Animal Welfare.

### Treatment Room, Laser, and Other Equipment

• Laminar flow hood (Baker Distributing Company, Kansas City, MO)
• Zeiss 30 SL customized slit-lamp laser delivery system (Carl Zeiss AG, Oberkochen, Germany)
• OcuLight GLx laser system (Iridex, Mountain View, CA)
• Laser safety goggles, 532–810 nm (Iridex)

### Reagents

All reagents were in sterile packages, and medication and drops were filtered to ensure sterility. They included the following:

• Ketamine hydrochloride solution 100 mg/mL (Dechra Veterinary Products, Overland Park, KS)
• Xylazine 20 mg/mL (AnaSed, Shenandoah, IA)
• Phenylephrine hydrochloride ophthalmic solution 2.5% (Paragon BioTeck, Portland, OR)
• Tropicamide ophthalmic solution 1% (Bausch & Lomb, Rochester, NY)
• GONAK hypromellose ophthalmic demulcent solution 2.5% (Akorn Pharmaceuticals, Lake Forest, IL)
• Tetracaine hydrochloride ophthalmic solution 0.5% (Bausch & Lomb)
• Balanced salt solution 15 mL (Alcon, Fort Worth, TX)
• Clidox-S solution (Pharmacal Research Laboratories, Waterbury, CT)

### Culture Media

• Sabouraud dextrose broth (General Laboratory Products, Yorkville, IL)
• Nutrient broth (General Laboratory Products)
• BBL Brain Heart Infusion prepared culture media (Benton, Dickinson and Company, Sparks, MD)
• Peptone (General Laboratory Products)

### Reverse Transcriptase Polymerase Chain Reaction

• Primers: 27F forward (5′-AGA GTT TCC TGG CTC AG-3′) and Rp2 reverse (5′-ACG GCT ACC TTG TTA CGA CTT-3′) (Integrated DNA Technologies, Coralville, IA)
• Powersoil DNeasy kit (QIAGEN Sciences, Germantown, MD)
• Agarose gel (Thermo Fisher Scientific, Waltham, MA)
• PCR mix (Takara Bio, Mountain View, CA)

### Immunostaining

• Rhodamine-labeled Griffonia Simplicifolia Lectin I (GSLI; Vector Laboratories, Burlingame, CA)
• Prolong Gold antifade (Thermo Fisher Scientific)

### Microscopy

• TCS STED laser scanning confocal microscope (Leica Microsystems, Wetzlar, Germany)
• Fiji Image J software (National Institutes of Health, Bethesda, MD)

### Consumables

All of the materials and consumables used during the procedure were sterilized, autoclaved, and filtered with 0.22-µm filters (for liquids) or presterilized in sealed packages. They included sterile gowns, sleeves, and gloves; sterile cloth and paper drapes; 1-, 5-, and 10-mL syringes; conical tubes; 1- and 2-mL collection tubes; sterile needles; tube racks; utility scissors; forceps; surgical tape; and coverslips (Thermo Fisher Scientific), as well as 0.22-µm filters (Sigma-Aldrich, St. Louis, MO), 0.1-mm-diameter zirconia/silica beads (BioSpec Products, Bartlesville, OK), and 1000-µL pipette tip boxes (Mettler-Toledo Rainin, Oakland, CA).

### Methods

Series of stepwise actions were defined meticulously to perform sterile retinal laser photocoagulation in GF C57BL/6J mice that were bred and maintained in sterile flexible film isolators at the University of Chicago GRAF. Following is a guideline and step-by-step protocol for sterile delivery of retinal laser in GF animals that sustains and confirms sterility throughout the duration of the study.

### Housing and Husbandry

GF C57BL/6J mice were inbred and kept on a 12/12-hour light/dark cycle. To ensure sterility of the GF colony, they were kept, bred, and maintained in sterile flexible film isolator housing (Class Biologically Clean, Madison, WI), under standard gnotobiotic protocols including, but not limited to, sterile water autoclaved at 250°F for 30 minutes; JL Rat and Mouse/Auto 6F 5K67 (LabDiet, St. Louis, MO) diet; and autoclaved pine shavings. After the procedure, the animals were relocated to a hermetically sealed, ventilated cage to prevent contamination of the rest of the colony. After 14 days, the animals were transferred to a sterile cage and sacrificed to harvest retinas for tissue processing and microscopy.

### Laser Treatment Protocol

This protocol is designed to be performed by a three-person team: a laser operator (LO) and two assistants. The first assistant (FA) works with the second assistant (SA) to prepare the materials and reagents required for the procedure. The following steps were taken to execute the experiments.

#### Slit-Lamp and Laser Delivery System Set-Up

The treatment room should be a laser certified location within the gnotobiotic facility to allow efficiency and prevent contamination. The location of the laser delivery system with relation to other instruments in the treatment suite must ensure enough space for maneuvering. We marked the floor using colored tapes to predetermine the correct position of the slit-lamp, laser delivery system, and adnexa. A 532-nm OcuLight GLx green laser (Iridex) and customized Zeiss 30SL slit-lamp were used. The operating parameters were set according to previously published methods,^19^ with some modifications: spot size of 50 µm, power of 150 mW, and duration of 100 ms for photocoagulation.

#### Sterilization of Laminar Flow Hood

All inner surfaces of the laminar flow hood are sprayed with chlorine dioxide solution comprised of one part Clidox-S base solution, three parts water, and one part Clidox-S activator. The solution is prepared before the procedure and can be used within 24 hours of preparation. After 2 minutes, the surfaces are wiped and dried. The surfaces are then resprayed and allowed to air dry. The chlorine dioxide solution is also used to spray and resanitize materials that must enter the sterile area after passing the laminar flow zone. The laminar flow motor must remain on during the entirety of the procedure.

#### Gowning and Work Station Set-Up

**Figure 1.**
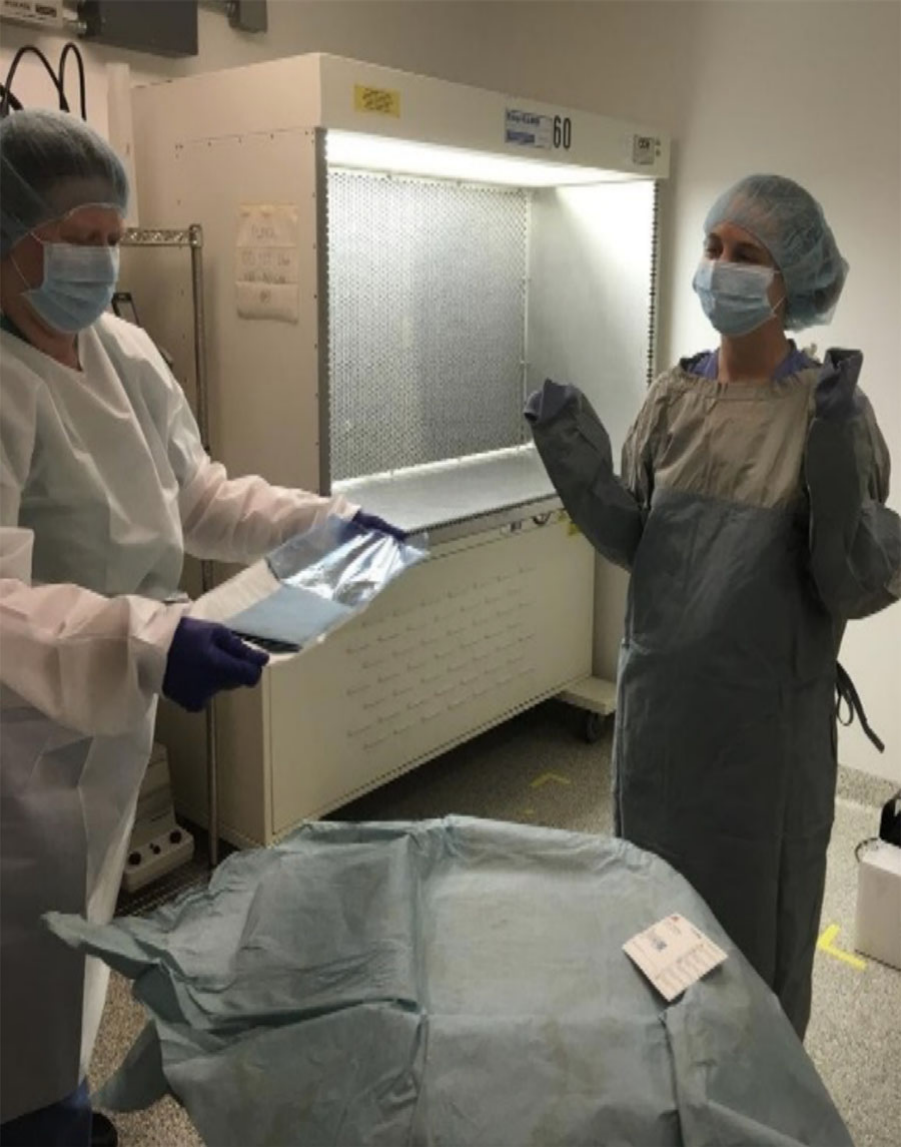
Gowning and sterile gloving. The SA (*left*) helps the FA (*right*) to gown up. The SA places the sterile gloves over a sterile surface, allowing the FA to put them on, minimizing surface contact.

**Figure 2.**
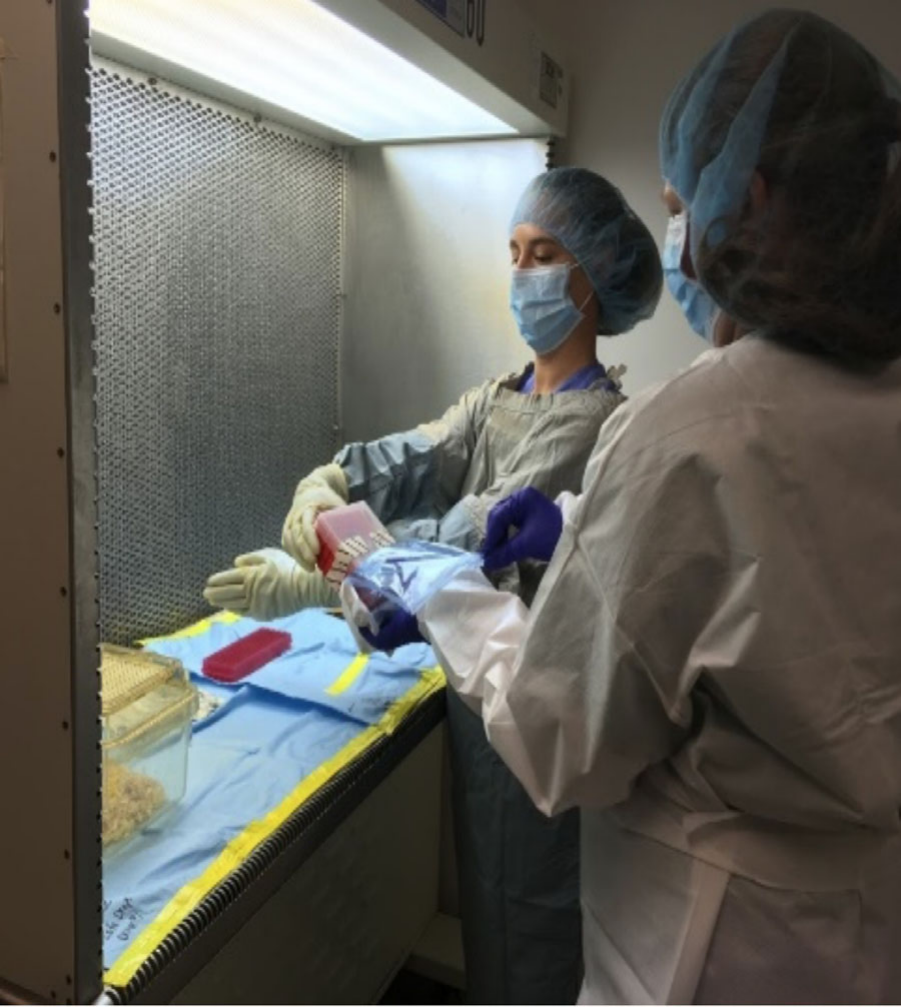
Material handling. The SA (*right*) must open the material wrapping so that only the sterile material is inside of the airflow and the FA's hand (*left*) does not have to exit the sterile area.

SA aids FA in putting on the sterile gown, sleeves, mask, hair net, and gloves, as shown in Figure 1. Sterile packages are opened and carefully handed to the FA. Double packaging is necessary. FA sets up the working area within the laminar flow hood. First, the working area is covered with sterile drapes fixed in place using tape. FA opens the secondary packages inside the work station, as depicted in Figure 2. The aliquoted reagents are placed in sterile racks ready for use. For the first experiments, aliquots can be prepared just before the experiment. The previously labeled sterile collection tubes are placed on a rack with their caps opened. Because most reagents cannot be sterilized using heat or chlorine gas, they are filtered using 0.22-µm disk filters. The collection tubes are placed in a-50 mL conical tube for storage and subsequent use. The treatment platform is placed at the edge of the laminar air curtain just within the area of laminar flow. We use a sterile pipette tip box as the platform, and the required height in our system is 3.6 inches. The platform is covered with a sterile cloth drape to prevent light reflection that might interfere with the visualization of the retina or laser procedure.

#### Animal Transfer and Preparation

**Figure 3.**
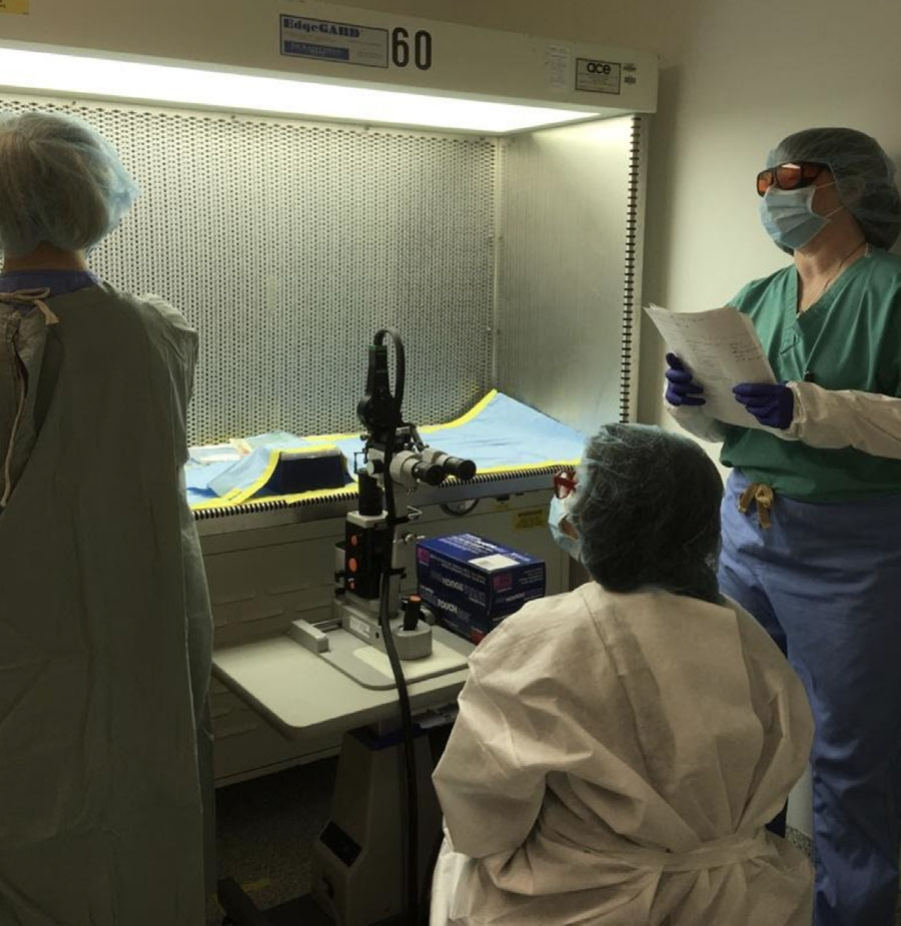
Laser operator and assistant positioning. The LO (*middle*) must be positioned just outside of the laminar flow hood. The FA is positioned at the left end of the laminar flow hood with forearms within the workspace to handle the mice while the SA stays at the right end of the hood to assist the operator and ensure proper execution of each step of the protocol by reading the steps to the FA and the LO.

SA leaves the treatment room, transfers the mice into the sterile cages, brings the mice to the treatment room, and facilitates sterile transport of animals under the hood via a sealed cylinder to avoid contamination. Each mouse is weighed using a gravitational scale, and the weight is used to calculate the anesthesia dose. The laser is set into position as depicted in Figure 3. LO gowns up with the help of SA. After entering the sterile area, LO cannot leave that position. Subsequently, SA assumes a position next to LO to ensure that the sleeve does not exit the laminar flow area. SA aids FA and anyone in the room in putting on laser protective goggles, which must remain on until the laser is no longer in use. Figure 3 illustrates the positioning of staff (LO, FA, and SA).

#### Anesthesia and Pupillary Dilation

**Figure 4.**
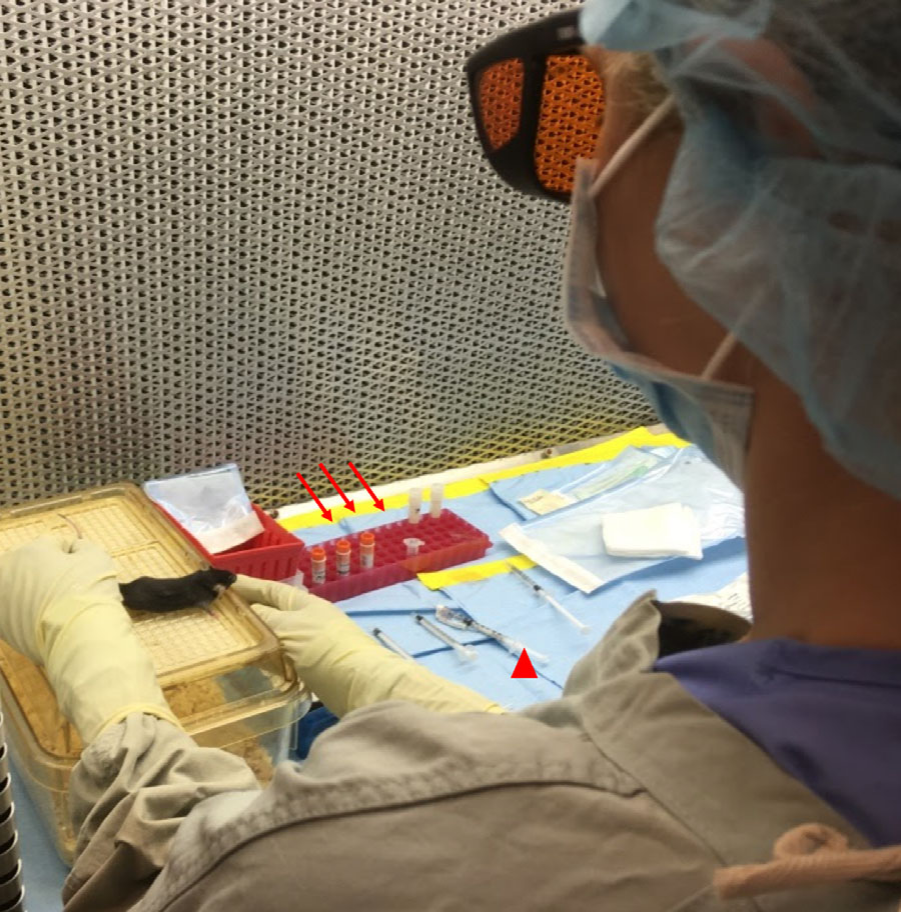
Anesthesia and preprocedural preparations and set-up. Laser protection goggles must remain on all assistants while the laser is operational. The tetracaine drop, anesthetics (ketamine/xylazine), tropicamide, and the sterile hypromellose ophthalmic demulcent solution are filtered, aliquoted, labeled, and secured on a rack (*red arrows*). The anesthesia cocktail is prepared and drawn in an insulin syringe (*red arrowhead*). After intraperitoneal injection of anesthetic medications, the FA confirms that the animal is not responsive by checking the response to stimuli, then proceeds to trim the whiskers with surgical scissors.

SA calculates the dosage of the anesthetic to be delivered for each subject. Ketamine 100 mg/kg and xylazine 10 mg/kg intraperitoneal are administered just before initiation of the laser treatment. FA applies to each eye of the mouse one drop of tetracaine solution, phenylephrine hydrochloride solution, and tropicamide solution to induce pupil dilatation. A drop of balanced salt solution (BSS) is used to keep the eyes moist. As soon as the animal becomes motionless, response to painful stimuli is checked; if absent, FA trims the whiskers and ensures that the eyes are wet with BSS drops at all times before the treatment, as shown in Figure 4.

#### Laser Treatment

**Figure 5.**
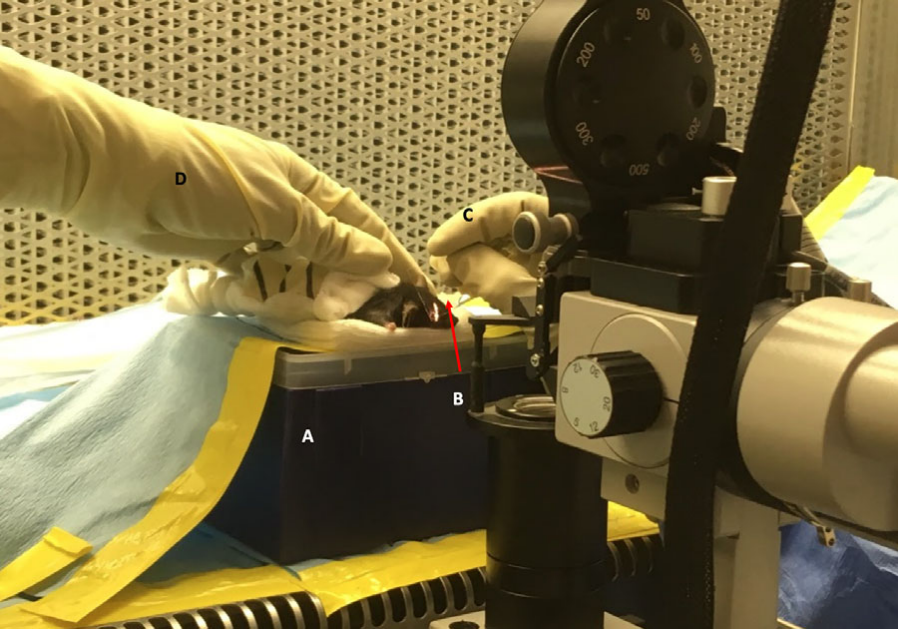
Animal positioning and laser treatment set-up. The mouse is placed on the platform (A), and the coverslip with BSS (B) is placed perpendicular to the laser beam over the eye before the laser procedure begins. The LO holds the coverslip in place with her/his right hand (C) and uses the left hand to handle the laser. The FA (D) helps to adjust the mouse's position to facilitate the procedure.

FA hands a glass coverslip with a BSS droplet on top to LO. LO places the coverslip over the eye perpendicular to the laser delivery port, then locates the optic nerve and aims the laser on the desired spot, focusing the aiming beam and activating a single shot. Figure 5 illustrates the animal positioning and laser platform set up. Four lesions are created using a power of 150 mW, a spot size of 50 µm, and a duration of 100 ms. The parameters can be adjusted to achieve the desired endpoint. The lesions are located at the 3, 6, 9, and 12 o'clock meridians centered on the optic nerve head and about 1 to 2 disc diameters from the optic nerve head. The presence of a bubble at the time of lasering is used as an indication of the rupture of Bruch's membrane. LO dictates where the spots are placed and whether or not laser treatment on each quadrant is successful (forming bubble, no immediate hemorrhage), and SA writes down this information. While the laser procedure takes place, FA prepares the next mouse for the procedure (as previously described in the Anesthesia and Pupillary Dilation section). When the treatment is done, the mouse that has received the laser treatment is switched with the new mouse. The tag number and specifications are announced by FA and written down by SA, and the laser treatment step is repeated.

#### Post-Treatment Monitoring and Transfer

After the treatment, mice recover on the hand warmer protected by sterile surgical stockinettes to avoid hypothermia. FA monitors the breathing rate during recovery, and when the animal has regained sternal recumbence it can be returned to the cage. LO leaves the hood area, de-gowns, and moves the slit-lamp laser delivery system away from the hood. SA hands to the FA a new set of autoclaved cages so each mouse can be placed in an individual sterile cage. After the procedure, the mice are kept in a single sterile, hermetically sealed cage ventilated system, and they are not placed back in the isolator to avoid any colony contamination. Chlorine dioxide solution is used to clean the hood, and a 70% ethanol solution is subsequently sprayed to remove any chlorine dioxide residues, as chlorine will corrode surfaces if not properly removed.

## Results

### Sterility Testing

**Figure 6.**
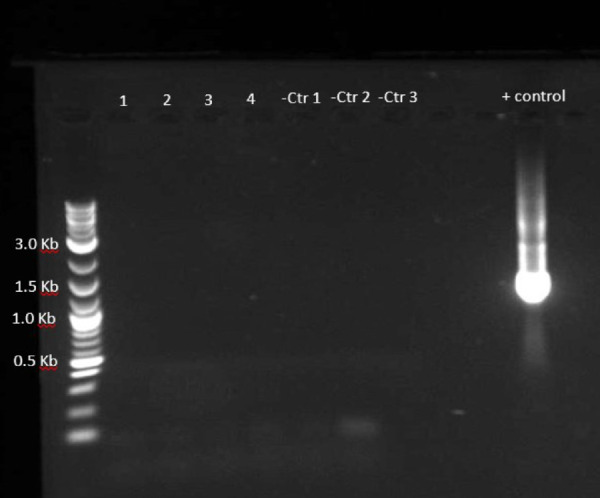
RT-PCR for 16s RNA. No amplification of 16s bacterial RNA was seen, confirming sterility in GF animals up to 2 weeks after the procedure. Fecal samples (1 and 3) were taken a week after the laser procedure from individually housed animals. Samples 2 and 4 were taken 2 weeks after laser procedure from individually housed animals. Negative control 1 (Ctr1), DNA from the fecal sample taken before the laser procedure; negative control 2 (Ctr2), PCR mix without DNA sample; negative control 3 (Ctr3), Milli-Q water; positive (+) control, DNA from the fecal sample taken from SPF mice.

We use two separate techniques to ensure sterility. Fecal pellets are collected prior to, during, and 7 and 14 days after laser treatment for culture as a conventional sterility test. Each week a fecal sample is taken from each cage for culture according to standard protocols as explained by Theriault et al.^40^ Per protocol, fecal pellets are used as inoculum in bacterial cultures. For each sample, multiple cultures are performed, including both aerobic and anaerobic at 37°C and aerobic at 42°C. These cultures are left for 5 days and monitored at 24, 48, 72, and 96 hours to observe for any growth. Reverse transcriptase–polymerase chain reaction (RT-PCR) is performed on the DNA of fecal samples for 16s rRNA as the confirmatory test. Total DNA extraction is performed using the QIAGEN PowerSoil DNeasy Pro Kit. PCR was performed with the primers 27F and Rp2 to obtain a 1500-bp product from bacterial 16s ribosomal DNA. In our study, seven GF mice in two separate experiment dates underwent the treatment. Stool cultures on days 0 were negative. All cultures (aerobic and anaerobic) from all seven animals (100%) remained negative at days 7 and 14 after the laser treatment. No amplification of 16s bacterial RNA was seen, confirming sterility in GF animals up to 2 weeks after the procedure. (Fig. 6)

### Confirmation of Choroidal Neovascularization Presence With Immunostaining

**Figure 7.**
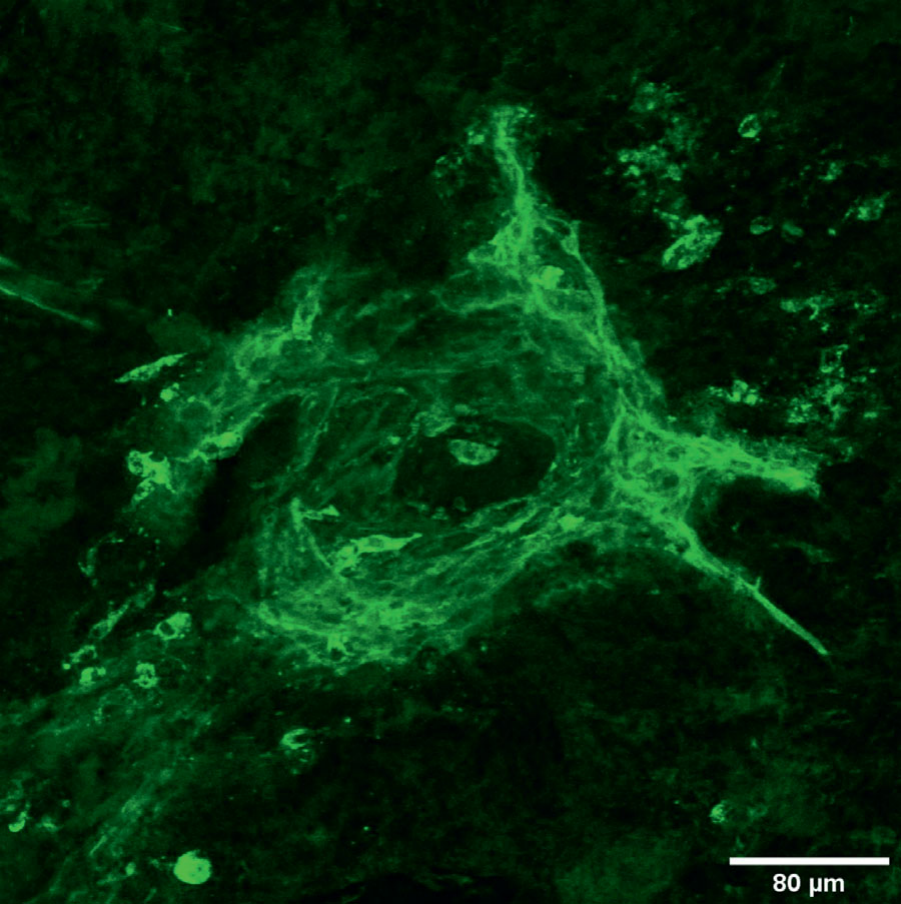
Immunostaining for lectin 1.20× confocal immunofluorescent image of a CNV lesion 2 weeks following laser treatment in a GF mouse, confirming successful laser delivery based on the presence of the CNV lesion.

To confirm successful laser delivery and formation of lesions, we performed lectin immunostaining 2 weeks after the laser procedure. Mice were euthanized using a CO_2_ chamber and cervical dislocation 14 days after the laser treatment. The eyes were fixed with 4% paraformaldehyde at 4°C. They were dissected, separating the choroid cup from the anterior segment, optic nerve, and retina. Retinas were harvested for histopathologic evaluation to confirm establishment of the model. Retinal whole (flat) mounts were prepared according to previously published protocols.^42^ Immunostaining was performed for histopathologic evaluation as previously described.^42^ Briefly, the cup was sectioned into four petals and then blocked overnight at 4°C with a donkey serum blocking solution (DSBS; 5% donkey serum, 2.5% BSA, and 5% Triton X-100 in 1× Tris-buffered saline [TBS-T]). The cups were transferred to a DSBS and rhodamine-labeled GSLI (1:400) for 2 hours at room temperature, washed six times for 10 minutes each with TBS-T, and then mounted on a glass slide using ProLong Gold antifade reagent which was sealed with a coverslip. The flatmount was left to dry at room temperature in the dark for 2 hours before examination, and the immunostained sections were imaged using a laser scanning confocal microscope. The images were processed using the Fiji Image J software. Lectin immunostaining confirmed successful delivery of the laser to the retinal tissue and development of laser-induced choroidal neovascularization, as shown in Figure 7.

## Discussion

A synergistic link between genetic predispositions and environmental factors, such as high-fat diet and severity of AMD, has been described.^43^^,^^44^ In the past few years investigators have looked more closely into the possible role of the gut microbiome in the pathogenesis of AMD.

Animal models are widely used in AMD research. The laser-induced CNV model is commonly used and accepted to study the mechanisms and treatments of neovascular AMD and ocular angiogenesis.^45^ GF animal models are considered the gold standard for studying host–microbe interactions in vivo but currently, there is no GF animal model of AMD. GF animals allow us to investigate the pure effect of the microbiome; by using this model, investigators have been able to describe the crucial role of the microbiome in inflammatory bowel disease, Parkinson's disease, allergic diseases, neoplasms, and the pathways involved in the gut–brain axis.^46^^–^^48^ One of the major benefits of the GF animals is that they can be colonized with known microbial flora to produce a specialized gnotobiotic animal model, based on a known microbiome composition associated with a particular disease.^19^^,^^22^^,^^26^

In this study, we defined a protocol to perform sterile retinal laser photocoagulation in GF mice, creating a GF version of the most commonly used mouse model of neovascular AMD. We were able to show the feasibility and sustained sterility throughout the duration of the study and up to 2 weeks after laser delivery. In developing this GF mouse model of CNV, we set out to demonstrate that a complex and extremely meticulous procedure of mouse retinal laser could be performed in a sterile fashion. Working with GF animals requires rigorous precautions with an overriding concern to prevent colony or individual contamination. Among the various types of study designs, interventional experiments on GF animals that require direct contact with the subjects carry the highest risk of contamination and may result in termination of experiments that often have been well planned and are extremely costly and time consuming. To solve this problem, many interventions, such as oral or topical treatments or even injections, could take place within sterile chambers where the investigator performs the intervention with at least one barrier layer between the animal and the researcher. Surgical interventions are among the treatments with the highest risk. Strategies to prevent contamination other than standard sterility measures may include surgical isolator use or performing procedures in a biological safety cabinet using strict aseptic and sterile techniques. None of these strategies was feasible for us, given the nature of the treatment for the CNV mouse model. The laser treatment involves a large apparatus for visualization and laser delivery and traditionally requires removal of mice from the cage and placing them on a special area of the slit-lamp chin rest within focus distance from the laser.^19^ Also, laser treatment is subject to specific laser safety guidelines and regulations at any institution with regard to the treatment room location, capacity, maintenance, and set-up, which complicate the process even more. The main challenges in this study were positioning the GF mice for the laser treatment and customization of the laser delivery system. To address these challenges, we placed the mice in a sterile laminar flow hood and modified the laser slit lamp so that the animal stayed inside the protected area of the sterile laminar air flow while the laser delivery apparatus got close enough to focus the laser beam on the fundus of the GF mice, as shown in Figure 5^6^,^7^.

The main goal and outcome of this study were to develop a detailed protocol demonstrating the sustainability of the GF laser-induced mouse model, which was confirmed by negative fecal cultures and confirmatory RT-PCR. The final outcome of the model was verified by the presence of CNV lesions in flatmount staining 2 weeks after the laser treatment. In this protocol, every step is crucial. The customized laser delivery system, sterility of the laminar flow hood, and following the detailed protocol precisely are of extreme importance. One major advantage of this protocol is that it can be modified easily to study other laser-induced ocular models such as retinal vein occlusion, optic neuropathy, glaucoma, laser retinopexy, ischemia–reperfusion injury, and wound healing. Additionally, the primary set-up can be used for diagnostic tools such as OCT, fluorescein angiogram, fundus photography, and electroretinography in GF mouse models.

Limitations of this procedure include the small number of animals tested so far and few repeats of the experiment. Because the focus of the study was to develop a GF laser CNV protocol and confirm the feasibility and maintenance of sterility—not to compare the CNV lesions in GF mice with the conventional mouse microbiome—we did not quantify the size of CNV lesions. Future studies analyzing the CNV lesions in GF are needed to address the mechanisms and effects of the microbiome on choroidal angiogenesis. Also, the CNV mouse model has its own set of limitations, the most distinct of which is that laser-induced CNV in mice does not exactly mimic the pathologic changes in human AMD.

To our knowledge, this is the first neovascular AMD model in GF mice. The development of this unique and valuable resource could represent an important milestone for AMD research toward answering fundamental questions about the role of the microbiome–retina axis and host–microbiome interactions in AMD. This GF animal model could pave the way toward a multitude of microbiome research studies utilizing gnotobiotic AMD animal models colonized with human microbiota, mouse fecal transplantation, or synthetic bacterial strains critical to investigate the role of gut microbiota in neovascular AMD and ocular angiogenesis. These experiments are now feasible and will allow us to delineate the mechanisms of how specific intestinal microbiota affect neovascular AMD/ocular angiogenesis and will lead to the discovery of new targets for therapeutic strategies by altering the gut microbiome with fecal transplantation of targeted microbiome-based compounds.

We believe this important animal model can become the gold standard tool for microbiome studies in vascular diseases of the eye, holding a promise of breakthrough microbiome research in ophthalmology in the future.
